# The mesenchymal stromal cell magic bullet finds yet another target

**DOI:** 10.1186/scrt471

**Published:** 2014-07-04

**Authors:** Claire Masterson, Daniel O’Toole

**Affiliations:** 1 Department of Anaesthesia, National University of Ireland Galway, University Road, Galway, Ireland; 2 Department of Medicine, National University of Ireland Galway, University Road, Galway, Ireland

## Abstract

Rojas and colleagues have presented an exciting paper demonstrating yet another relevant preclinical setting in which the mesenchymal stromal cell has a potential therapeutic application. What is particularly interesting about this study is that it addresses a disease, blood-borne systemic sepsis, which has multiple complex host responses and involves a variety of disparate organs and immune cell types. Here, the authors focus on how this injury relates more specifically to the lung, with quite dramatic improvements in several assessed injury parameters. Where does this latest demonstration of mesenchymal stromal cell efficacy leave us with regard to getting these cell therapies to the acute respiratory distress syndrome patient?

## 

The mesenchymal stromal cell (MSC) as a proposed treatment for acute respiratory distress syndrome (ARDS) appears to be rapidly approaching the moment of truth for any new drug. We have the disease, we have the medicine, and we have the preclinical data – now for the important part. Rojas and colleagues’ paper informs us of another possible application of the MSC: treatment of ARDS as a consequence of a systemic infection [1].

Of note, especially in the acute clinical setting of ARDS, is the fact that this MSC is administered directly from cryopreservation. There has been much recent debate as to whether a wake-up period is essential for the beneficial effects seen with MSCs in injury models, and it is heartening to see that an off-the-shelf product such as MultiStem (Athersys, Cleveland, OH, USA) can be used in a rapid, multiorgan failure setting such as sepsis. This observation is of enormous importance because there are now up to 10 years of promising findings in models that have utilized freshly harvested passaging MSCs, with some trepidation as to whether a −150°C freezer stock of MSCs in the intensive care department was ever likely to be the eventual outcome. While confirming some findings [2], this is also a direct contradiction of some earlier work that failed to demonstrate adequate therapeutic potential of MSCs immediately after thaw from cryostorage [3].

There are, however, still some concerns and possible missing pieces in this jigsaw leading to a picture of a perfected translation to the clinic. Firstly, this study utilizes bacteria-derived lipopolysaccharide as the injurious agent. Whilst this recapitulates many of the host inflammatory responses in a sepsis setting, it does not address the additional necessity of bacteria clearance from the host. While antibiotics may go some way towards alleviating this concern, an injury model utilizing clinically isolated pathogens with administration of a relevant broad-spectrum antibiotic would raise confidence immensely in the MultiStem product’s real-world efficacy. Indeed, recent work has begun to address the direct antibacterial effects of the MSC alongside its ability to enhance the function of macrophages and perhaps other leukocyte populations [4,5]. Furthermore, and while not a comment directed at this study of systemic sepsis, the persistence of *Escherichia coli* (or its products) as the preferred causative of pneumonia in lung injury research is perhaps occluding the reality of multiple, indeed often unidentified, pathogen involvement in the aetiology of pneumonia-induced ARDS.

Another caveat is that while in certain aspects it is appealing due to possible enhanced safety and a lower required dose of MSCs, intratracheal (or in this case the even more invasive intrabronchial) administration of any appreciable liquid volume of therapeutic may be a tall order in an established ARDS patient due to the excess fluid infiltration into the lung air space. Adding more liquid to an already poorly performing lung may be a risk the physician is unwilling to take. Intratracheal/intrabronchial studies offer a wealth of information with regards to mechanism of action, but the success thus far of numerous studies using the intravenous route would appear to make this the most promising with respect to ultimate delivery to the patient.

Despite this, preclinical validation of any possible therapeutic for the devastating condition of sepsis/ARDS is to be welcomed, and we look forward intently to following up the MultiStem MSC as it forges it way towards success in the true clinical setting.

## Abbreviations

ARDS: Acute respiratory distress syndrome; MSC: Mesenchymal stromal cell.

## Competing interests

The authors declare that they have no competing interests.

## Authors’ contributions

The authors contributed equally to the preparation, writing and proofreading of this manuscript. Both authors read and approved the final manuscript.
